# Case Report: Japanese Siblings of Cystic Fibrosis With a Novel Large Heterozygous Deletion in the *CFTR* Gene

**DOI:** 10.3389/fped.2021.800095

**Published:** 2022-01-03

**Authors:** Mayumi Kawase, Masato Ogawa, Takayuki Hoshina, Masumi Kojiro, Miyuki Nakakuki, Satoru Naruse, Hiroshi Ishiguro, Koichi Kusuhara

**Affiliations:** ^1^Department of Pediatrics, Kitakyushu General Hospital, Kitakyushu, Japan; ^2^University of Occupational and Environmental Health, Kitakyushu, Japan; ^3^Department of Human Nutrition, Nagoya University Graduate School of Medicine, Nagoya, Japan; ^4^Department of Internal Medicine, Miyoshi Municipal Hospital, Miyoshi, Japan

**Keywords:** cystic fibrosis, novel variant, promoter region (genetics), multiplex ligation-dependent probe amplification (MLPA), *ASZ1* gene

## Abstract

Cystic fibrosis (CF) is a rare disease in the Japanese. The most common *CFTR* variant in Japanese CF patients is a large heterozygous deletion that can easily avoid detection by standard gene sequencing methods. We herein report a novel large heterozygous deletion in the *CFTR* gene in Japanese siblings with CF. A genetic analysis was performed in two patients (9-year-old boy and 5-month-old girl) who were clinically diagnosed with CF because of the positive result for the rapid fecal pancreatic elastase antigen test and the elevation of the sweat chloride concentration. In addition to conventional polymerase chain reaction (PCR) and direct sequencing, multiplex ligation-dependent probe amplification (MLPA) was performed to check for a large deletion and duplication of the *CFTR* gene. Based on MLPA findings, the breakpoint of heterozygous deletion was identified by real-time quantitative PCR followed by the sequence of the amplified junction fragment. In MLPA, the numbers of the fragments corresponding to exons 1, 16, 17a, and 17b and 234 nt and 747 nt upstream from the translation initiation codon of exon 1 in the *CFTR* gene and exon 3 in the *ASZ1* gene were reduced by almost half. The c.2908+1085_3367+260del7201 variant (exon 16-17b deletion) was identified in one allele. The other allele had a large 137,567-bp deletion from g.117,361,112 (*ASZ1* 3′ flanking region) to g.117,498,678 (*CFTR* intron 1) on chromosome 7. Since the deletion variant lacked the entire promoter region of *CFTR, CFTR* mRNA would not be transcribed from the allele, indicating it to be a novel pathogenic variant causing CF. As large mutations are frequently detected in Japanese CF patients, MPLA can be useful when searching for variants.

## Introduction

Cystic fibrosis (CF) is an autosomal recessive genetic disorder that is common in Caucasian population with an estimated incidence of 1 in 3,500 newborns. CF is considered to be very rare in Japan, with an incidence of ~3 per 1 million individuals (1). However, this incidence of CF may be underestimated, as the sweat chloride test required for the definite diagnosis is not readily available, and the *CFTR* mutations identified in Japanese patients are typically rare in Caucasians (2–4). The most common variant in Japanese CF patients is a large heterozygous deletion that can easily evade detection by standard gene sequencing methods (3, 4).

We herein report Japanese siblings diagnosed with CF who had a novel large heterozygous deletion in the *CFTR* gene, in addition to the previously-known large deletion.

## Case Presentation

### Case 1

A 9-year-old Japanese boy who was the first child of non-consanguineous Japanese parents was admitted to our hospital for the treatment of acute bronchiolitis. The patient had been receiving oral and inhalant medications for the treatment of bronchial asthma since 6 years old, but the medications were discontinued at his mother's discretion 6 months before admission. He had been hospitalized for unexplained weight loss, hyponatremia and hypochloremia at 7 months of age. His maternal grandfather had lung cancer and his mother had bronchial asthma.

**Figure 1:**
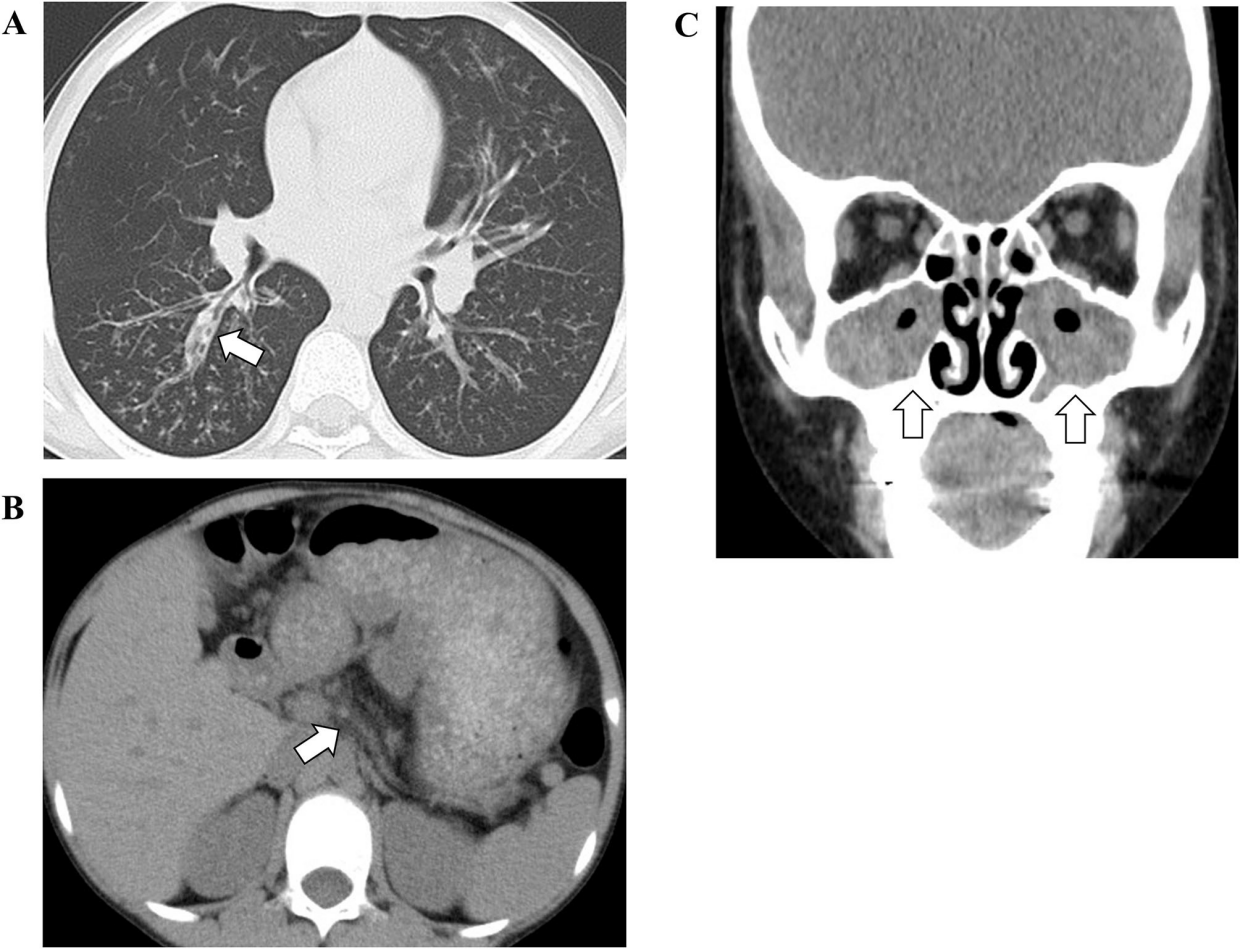
The findings of chest **(A)**, abdominal **(B)**, and facial **(C)** computed tomography. The white arrow in **(A)** indicates thickened bronchial walls and bronchiectasis. The white arrow in **(B)** indicates an atrophic pancreas. The white arrows in **(C)** indicate gas-fluid level in the paranasal sinuses and gas bubbles within the fluid.

On admission, he had a low-grade fever (37.9°C) and oxygen desaturation (surface oxygen saturation 89%). Rhonchi were heard on auscultation. Chest X-ray showed thickened bronchial walls around the bilateral pulmonary hilar regions, and the chest computed tomography (CT) showed hyperinflation of the lung field, thickened bronchial walls and bronchiectasis (Figure 1A). In addition, an atrophic pancreas with fatty infiltration was incidentally detected on CT (Figure 1B). He was diagnosed with sinusitis based on the gas-fluid level in the paranasal sinuses and gas bubbles within the fluid on the CT (Figure 1C).

CF was suspected based on these clinical findings and imaging. A low serum trypsin level [52 ng/mL, reference range (rr) 100–550], decreased insulin secretion (insulin secretory index 0.17, rr > 1.10) and positive result for the rapid fecal pancreatic elastase antigen test indicated the pancreatic exocrine and endocrine insufficiency. The sweat chloride concentration was elevated (120 mmol/L, rr < 40). Based on these findings, he was clinically diagnosed with CF.

### Case 2

A 5 month-old girl who was a younger sister of case 1 visited our hospital because of poor feeding and weight gain. Her development was age-appropriate. The physical examination revealed almond-shaped eyes and small, unbalanced extremities. The serum levels of electrolytes were normal. The rapid fecal pancreatic elastase antigen test was positive. The sweat chloride concentration was 120 mmol/L. The patient was also clinically diagnosed with CF.

## Materials and Methods

### Direct Sequence of the CFTR Gene

Genomic DNA was extracted from peripheral leukocytes. As reported in a previous study (4), Polymerase chain reaction (PCR) was performed, and all 27 exons as well as their boundaries (100–300 bp including poly T and TG repeats in intron 8) and the promoter region (up to 1,028 bp upstream of the translation initiation codon of exon 1) of the *CFTR* gene were directly sequenced.

### A Quantitative Fragment Analysis by Multiplex Ligation-Dependent Probe Amplification

MLPA, a quantitative PCR-based assay (5), was performed to check for the presence of a large deletion and duplication of the *CFTR* gene (6). The SALSA P091-C1 CFTR MLPA kit (MRC Holland, Amsterdam, Netherlands) was used. The kit contains probes for each of the 27 exons of the *CFTR* gene. Fragments were separated on CEQ-8000 capillary electrophoresis system (Beckmann Coulter, Fullerton, CA, USA) using the Beckmann D1-labeled CEQ size standard 600.

### Detection of the Mutation of c.2908+1085_3367+260del7201 in the CFTR Gene

We attempted to detect the mutation of c.2908+1085_3367+260del7201 (exon 16-17b deletion) in one allele of the *CFTR* gene using the method to amplify the junction fragment (4). The primers were set on introns 15 and 17b to amplify the deleted allele.

### The Analysis of the Genomic Rearrangement of the CFTR Gene

To identify the deletion breakpoints of the novel large deletion spanning *CFTR* exon 1 and *ASZ1*, we first narrowed down the breakpoint site by repeating real-time Quantitative PCR (7), which was designed to distinguish the site of heterozygous deletion by comparing cycle threshold (Ct) values defined as the number of cycles required for the fluorescent signal to cross the threshold. Concentrations of DNA extracted from blood samples collected from Case 1 and a healthy adult (control subject) were adjusted at ~20 ng/μL. The real-time PCR was carried out using these samples and the Ct-values of the assay between Case 1 and the control subject were compared. The ΔCt-value that was determined by subtracting the Ct-value of the control subject from that of Case 1 was calculated for the comparison of the two samples. The first round of real-time PCR was carried out at ~4kb intervals within *CFTR* intron 1 (3′-side) and downstream of *ASZ1* exon 3 (5′-side). The second and third rounds of real-time PCR were carried out at ~200 bp. The list of primers is shown in Table 1. Primers were added at a final concentration of 0.4 μM with TB Green Premix Ex Taq II (Takara Bio, Otsu, Japan). The assay was run in a M × 3005P Real-time QPCR system (Stratagene's An Agilent Technologies Division, La Jolla, CA, USA). The exact delineation of the deletion was carried out by PCR amplification of the junction fragment harboring the breakpoint (primers are noted in bold in Table 1) and sequence of the PCR products. The PCR products were purified and cloned using the Mighty TA-cloning Kit (Takara Bio) and sequenced directly.

**Table 1:** Primers for real-time quantitative polymerase chain reaction.

**No**.	**Location**	**g number of 5'end^a^**		**Sequence**	**Amplicon**	**Ct^b^**	**Ct^b^**	**ΔCt^c^**
					**length (bp)**	**(case 1)**	**(control)**	
1	*WNT2* exon2	117,315,131	F	5′-TTCCTTTCCTTTGCATCCAC	213	26.90	26.57	0.33
	*WNT2* exon2	117,315,343	R	5′-CGGGAATCTGCCTTTGTTTA				
2	*WNT2* intron 1	117,320,515	F	5′-GAGCTGTGCATGAGTGGAGA	249	26.81	26.86	−0.05
	*WNT2* intron 1	117,320,763	R	5′-GGTGATGTGCGATAATGTGC				
3	*WNT2* intron 1	117,322,739	F	5′-AAAGAGAAGGGGCTCACCAT	218	27.10	26.83	0.27
	WNT2 exon 1	117,322,956	R	5′-CTCCCTCTGCTCTTGACCTG				
4	***ASZ1*** **3′** **flanking region**	**117,360,440**	**F**	**5** **′** **-TGCATGAGTGCTGGAAAGAG**	239	23.64	23.64	0.00
	*ASZ1* 3′ flanking region	117,360,678	R	5′-AGGCTCAGGACAGAGATGGA				
5	*ASZ1* 3′ flanking region	117,361,181	F	5′-CTATAGGTTGGTGGGCCAAA	158	24.92	23.90	1.02
	*ASZ1* 3′ flanking region	117,361,338	R	5′-TTTGCAGGACATGTGGTCTC				
6	*ASZ1* 3′ flanking region	117,362,771	F	5′-TGGCACTAAGTCAGGCAAGA	168	24.89	23.96	0.93
	*ASZ1* 3′ flanking region	117,362,938	R	5′-AAAGTGAATGGCATTTGACATAT				
7	ASZ1 3′ UTR	117,363,536	F	5′-GCAATGATTTTTGGATGGTTC	211	26.49	25.64	0.85
	*ASZ1* exon 13	117,363,746	R	5′-GCAAAATGAACGGGAAAATG				
8	*ASZ1* intron 12	117,367,167	F	5′-GCATTTGCTTAATGGCCAAC	163	24.80	23.90	0.90
	*ASZ1* intron 12	117,367,329	R	5′-AGGGGAAAAATGAAGGGAAA				
9	*ASZ1* intron 10	117,379,821	F	5′-TTACCCTGGGAAAATGTGG	204	26.40	25.10	1.30
	*ASZ1* exon 10	117,380,024	R	5′-GCAGAAAATTCTGGCTGCTC				
10	*ASZ1* exon 9	117,381,020	F	5′-TTCATCTTCCCTCATGGTCA	174	27.00	25.80	1.20
	*ASZ1* intron 8	117,381,193	R	5′-TGTCCCCTCTAGAAAATTGGAA				
11	*ASZ1* intron 8	117,381,502	F	5′-AAACACCCACACAGTGCTTG	350	25.90	24.70	1.20
	*ASZ1* intron 8	117,381,851	R	5′-TTACCCACTGCCTAACCTTCA				
12	*ASZ1* intron 6	117,384,571	F	5′-CATTACCTGGCTGGAGGAAA	193	28.00	27.00	1.00
	*ASZ1* exon 6	117,384,763	R	5′-TGCCAAGTGAGATTGCAAAA				
13	*ASZ1* exon 5	117,385,755	F	5′-AACAACCTGGGTGTGACCAT	216	26.19	25.50	0.69
	*ASZ1* intron 4	117,385,970	R	5′-TGCAGGAAGGTTGGATTTTT				
14	*ASZ1* intron 4	117,419,068	F	5′-GGTTTCTCAACCATGGCACT	178	21.81	20.78	1.03
	*ASZ1* intron 4	117,419,245	R	5′-CAGGGGTATTTGGCAATGT				
15	*CFTR* intron 1	117,480,042	F	5′-TTAGGAGCTTGAGCCCAGAC	171	23.83	23.10	0.73
	*CFTR* intron 1	117,480,212	R	5′-CATACACACGCCCTCCTCTT				
16	*CFTR* intron 1	117,486,707	F	5′-AGATCGTAAGGGGGCTTTGT	153	23.71	22.89	0.82
	*CFTR* intron 1	117,486,859	R	5′-GGCCACTCTTTCAGCTCATC				
17	*CFTR* intron 1	117,488,982	F	5′-GCATCCCACAAGGTTGACTC	165	24.68	23.94	0.74
	*CFTR* intron 1	117,489,146	R	5′-GTGCTGAGCTTAGGCGACTT				
18	*CFTR* intron 1	117,490,429	F	5′-CAGGAAACCCAGGAGAGTCA	286	24.55	23.73	0.83
	*CFTR* intron 1	117,490,714	R	5′-CGCCCTATGTCTGGCATTAT				
19	*CFTR* intron 1	117,492,959	F	5′-CCATGCCCAGTGATGGTAAT	122	23.61	22.62	0.99
	*CFTR* intron 1	117,493,080	R	5′-AACGCTGTGCCAGATTCTCT				
20	*CFTR* intron 1	117,495,294	F	5′-ATCCTGGAAAGGCACTCTGA	102	23.68	22.72	0.96
	*CFTR* intron 1	117,495,395	R	5′-ATCCCACCCATCTTGAAACA				
21	*CFTR* intron 1	117,495,476	F	5′-GCTGTTAGAAGTGGCCTTTCA	233	24.59	23.62	0.97
	*CFTR* intron 1	117,495,708	R	5′-GGGTGGCTACAGCAAGTGAT				
22	*CFTR* intron 1	117,497,112	F	5′-GGCTTTGGTGTCACAATCCT	141	24.56	23.69	0.87
	*CFTR* intron 1	117,497,252	R	5′-TGATCCCCACAACAATTCAA				
23	*CFTR* intron 1	117,497,521	F	5′-GAGCTTTTTCCAAGGCGATA	140	25.05	23.83	1.21
	*CFTR* intron 1	117,497,660	R	5′-TACGAATCCCCAGTCACCTG				
24	*CFTR* intron 1	117,498,301	F	5′-AGGCTTGTCTTTAGCGAGCA	158	24.98	24.09	0.89
	*CFTR* intron 1	117,498,458	R	5′-CGCAGTATTGGGGTCAAGTT				
25	*CFTR* intron 1	117,499,022	F	5′-TTTGGGAGAAGTGTCATGCA	213	24.95	24.69	0.25
	***CFTR*** **intron 1**	**117,499,234**	**R**	**5** **′** **-TCCAAAAGACGCATCTGACA**				
26	*CFTR* intron 1	117,499,949	F	5′-GTGAGAGGGGAAGACAGCAG	207	23.43	23.61	−0.18
	*CFTR* intron 1	117,500,155	R	5′-ACTCCAGCCACCCTTTCTTT				
27	*CFTR* exon 1	117,504,160	F	5′-TTCCATATGCCAGAAAAGTTGA	140	23.66	23.89	−0.23
	*CFTR* exon 2	117,504,299	R	5′-ATTCGAGGCGCTGTCTGTAT				

a *GRCh38.p12; NC_000007.14, F: forward, R: reverse*.

b *Ct-values are defined as the number of cycles required for the fluorescent signal to cross the threshold in the real-time quantitative polymerase chain reaction*.

c *The ΔCt-value is determined by subtracting the Ct-value of the control subject from that of Case 1*.

*Ct, Cycle threshold*.

## Results

**Figure 2:**
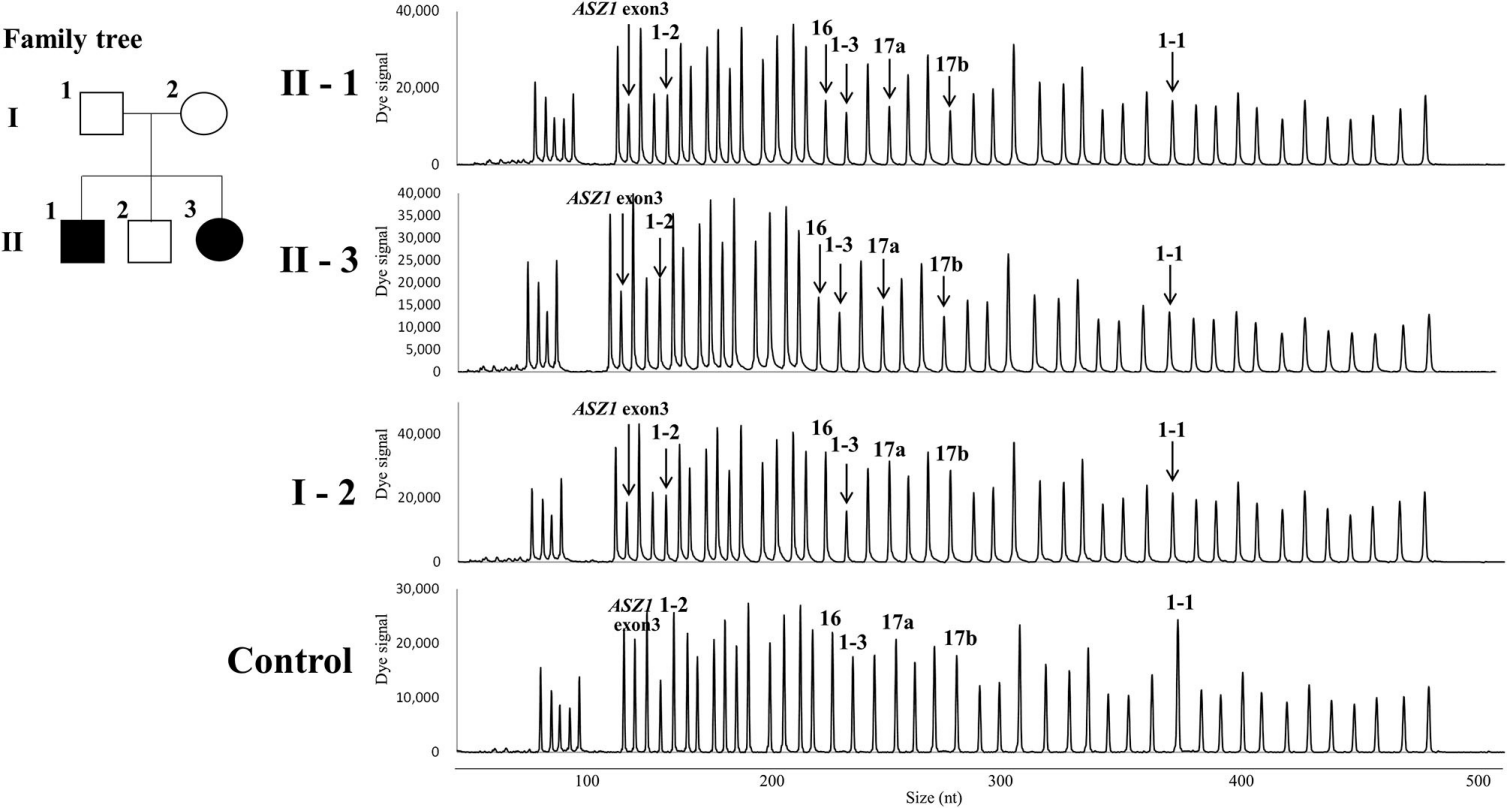
Family tree of the patients and the quantitative fragment analysis by multiplex ligation-dependent probe amplification. 1-1, 1-2, 1-3, 16, 17a, and 17b indicate the signals corresponding 747 nt upstream the translation initiation codon, 234 nt upstream the translation initiation codon, exons 1,16,17a, and 17b in the *CFTR* gene, respectively. The down-pointing arrows indicate the reduction of signals.

Direct sequencing of the *CFTR* gene detected no previously-recognized mutations related to the development of CF in either patients. Next, we performed a quantitative fragment analysis by MLPA. In the samples from cases 1 and 2, the numbers of fragments corresponding to exons 1, 16, 17a, and 17b and 234 nt and 747 nt upstream from the translation initiation codon of exon 1 in the *CFTR* gene and exon 3 in the *ASZ1* gene were reduced by almost half, compared with that from the control (Figure 2). In the sample from their mother, 234 nt and 747 nt upstream from the translation initiation codon of exon 1 in the *CFTR* gene and exon 3 in the *ASZ1* gene were reduced by almost half (Figure 2). These data indicated that there were large heterozygous deletions in both alleles of the *CFTR* gene in cases 1 and 2. One was probably the deletion of exon 16-17b inherited from their father, and the other was a novel large deletion spanning *CFTR* exon 1 and *ASZ1* inherited from their mother. PCR amplification of the junction fragment identified the deletion of exon 16-17b (4) in one allele of cases 1 and 2 (data not shown). We could not obtain the samples from patients' father because of his disapproval.

**Figure 3:**
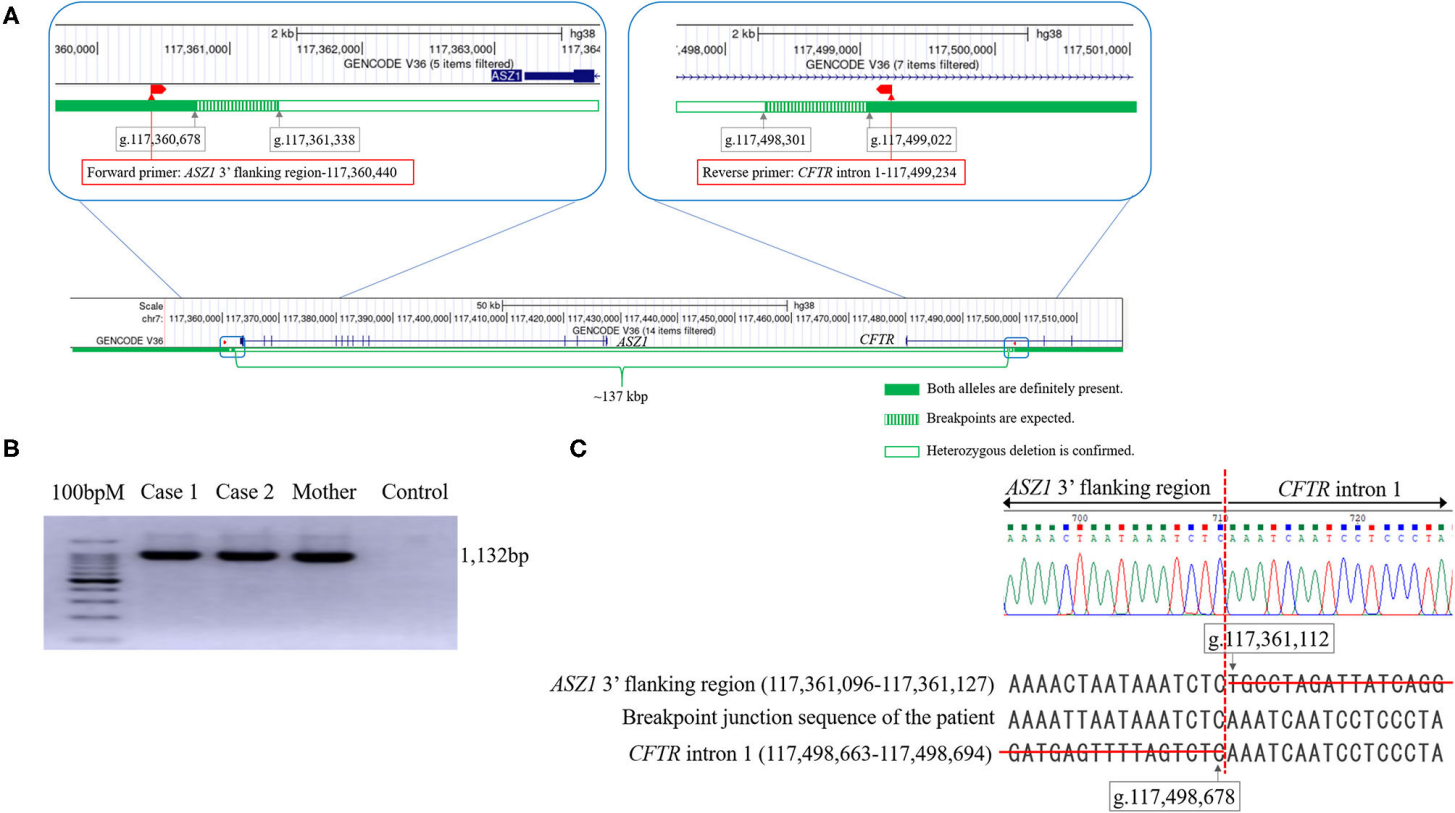
The analysis of the genomic rearrangement of the large deletion spanning *CFTR* exon 1 and *ASZ1*. **(A)** Sequencing results of the large deletion (~137 kbp). Exon 1 and the promoter region of the *CFTR* gene and all of the *ASZ1* gene are included in this large deletion (*CFTR* promoter deletion). According to the data of real-time quantitative PCR, the 5′- and 3′- breakpoints of the deletion were expected to be within green hatched regions. The locations of forward and reverse primers to amplify the junction fragment are indicated as red boxes. The figures of genomic structure analysis were adapted from UCSC Genome Browser (GRCh38/hg38). **(B)** Agarose gel electrophoresis of the junction fragment. Cases 1 and 2 and their mother have the large deletion. **(C)** Sequencing results of the junction fragment obtained from the genomic DNA reveals a large 137,567 bp deletion from g.117,361,112 (*ASZ1* 3′ flanking region) to g.117,498,678 (*CFTR* intron 1) on chromosome 7.

Real-time quantitative PCR demonstrated that the ΔCt was ~1 (the copy number of DNA in case 1 was two-fold lower than that in control) in reactions with primers of Nos. 5–24 (Table 1). Thus, the deletion breakpoints of the novel large deletion spanning *CFTR* exon 1 and *ASZ1* were expected to be within g.117,498.301 to g.117,499,022 (3′-side) and g.117,360,678 to g.117,361,338 (5′-side) (Figure 3A). PCR of the junction fragment amplified the 1,132-bp product from case 1 and 2 and their mother (Figure 3B). Sequence of the PCR products revealed a large 137,567-bp deletion from g.117,361,112 (*ASZ1* 3′ flanking region) to g.117,498,678 (*CFTR* intron 1) on chromosome 7 (Figure 3C). Exon 1 and the promoter region of the *CFTR* gene as well as all of the *ASZ1* gene were included in this region. Since the deletion variant lacked whole of the promoter region of *CFTR, CFTR* mRNA would not be transcribed from the allele. This deletion (GRCh38.p12:NC_000007.14:g.117,361,112_117,498,678del137, 567) was thus regarded as a novel pathogenic variant causing CF.

## Discussion

We encountered Japanese siblings with CF who had a novel variant (*CFTR* promoter deletion) in addition to exon 16-17b deletion, that is the most common *CFTR* variant in Japanese CF patients (3, 4). The exon 16-17b deletion causes a deletion of 153 amino acids, p.(Gly970_Thr1122del)-CFTR, that is located over 3 transmembrane helices of the CFTR protein (3). p.(Gly970_Thr1122del)-CFTR was synthesized but not transported to the membrane when expressed in CHO cells, indicating that it can be categorized as a class II mutation (3, 8). The novel variant, *CFTR* promoter deletion, is not be transcribed due to the lack of whole of the promoter region of the *CFTR* gene and thus can be categorized as a class I mutation (8).

CF is a multiorgan disease with marked phenotypic heterogeneity. The pathogenic *CFTR* variants are grouped into five classes according to the mechanism by which the variants affects the normal CFTR protein function (8). Patients who are homozygous or compound heterozygous for the severe variants belonging to classes I, II and III are underweight, more often required pancreatic enzyme supplementation and have a higher prevalence of diabetes than patients carrying at least one class IV mutation (9). Among CF patients, pulmonary disease is the main cause of death, and the colonization of *Pseudomonas aeruginosa* in the respiratory tract is associated with a reduced respiratory function and increased pulmonary exacerbation (10, 11). Severe variants are also linked to a high probability of the colonization of *P. aeruginosa* in the respiratory tract (12). The present patients were compound heterozygous for the severe variant (class I/class II) and were immediately prescribed inhalation of recombinant human deoxyribonuclease I and replenishment of pancreatic enzyme after receiving their diagnosis. Fortunately, the respiratory function has not worsened in either of these patients, and *P. aeruginosa* has been never isolated from respiratory tract. The early therapeutic intervention after the diagnosis of CF helps protect the lungs from damage and improves the prognosis.

As the life expectancy of CF patients continues to increase, infertility has become a critical problem in these patients. Nearly all men with CF had infertility due to obstructive azoospermia (13), specifically congenital bilateral absence of the vas deferens, which is mainly caused by the variant in the *CFTR* gene (14). In our patients, the *ASZ1* gene encoding *ASZ1*, which is only expressed in the testis and ovary and probably plays a role in germ cell development (15), was also completely deleted on the one allele. Either the deletion of *Asz1* or the variant leads to male infertility in mice (16). No CF patients have shown the deletion of the *ASZ1* gene. Male CF patients produce sperm, and their testicular histology is usually normal (17). Although the *ASZ1* gene was deleted only on the one allele in our patients, the analysis of sperm in case 1 may allow us to investigate the association between the *ASZ1* gene and male infertility in humans.

As in the present sibling cases of CF, large heterozygous deletions escape detection by standard gene sequencing methods. In contrast to Caucasians, such large deletions are frequently detected in Japanese CF patients (3). MLPA can detect all deletions, not just the common one. Thus, in Japanese CF patients, MPLA can be more useful in searching for variants of *CFTR* gene.

## Data Availability Statement

The original contributions presented in the study are included in the article/supplementary material, further inquiries can be directed to the corresponding author/s.

## Ethics Statement

The studies involving human participants were reviewed and approved by Institutional Review Boards of Kitakyushu General Hospital and Nagoya University Graduate School of Medicine. Written informed consent to participate in this study was provided by the participants' legal guardian/next of kin.

## Author Contributions

MK involved the treatment of patients, carried out the initial analysis of data for work, and drafted the initial manuscript. TH and KK carried out the acquisition and analysis of data for the work and revised the work critically for important intellectual content. MO and MK involved the treatment of patients and carried out the acquisition and analysis of data for the work and revised the work critically for important intellectual content. MN, SN, and HI carried out the analysis of data for the work and revised the work critically for important intellectual content. All authors contributed to the article and approved the submitted version.

## Conflict of Interest

The authors declare that the research was conducted in the absence of any commercial or financial relationships that could be construed as a potential conflict of interest.

## Publisher's Note

All claims expressed in this article are solely those of the authors and do not necessarily represent those of their affiliated organizations, or those of the publisher, the editors and the reviewers. Any product that may be evaluated in this article, or claim that may be made by its manufacturer, is not guaranteed or endorsed by the publisher.
